# Analysis of data and common mutations encountered during routine parentage testing in Zimbabwe

**DOI:** 10.1038/s41598-024-51987-8

**Published:** 2024-01-16

**Authors:** Roslyn Stella Thelingwani, Catherine Ashley Jonhera, Collen Masimirembwa

**Affiliations:** 1 Forensic Science Unit, Department of Genomic Medicine, African Institute of Biomedical Science and Technology (AiBST), 911 Boronia Township, Beatrice, Zimbabwe; 2 CradleOmics, Block C, Wilkins Hospital Complex, Harare, Zimbabwe

**Keywords:** DNA, Genetics, Molecular biology

## Abstract

We analyzed parentage data collected over a ten-year period in a Zimbabwean DNA testing laboratory. Parentage case types, prevalence, exclusion data, mutations rates and observed genotyping irregularities were analyzed. We report analysis results from 1303 cases. DNA extraction and STR typing was conducted using standard commercial kits. Paternity was the most requested test (87.37%) followed by the indirect biological kinship tests (7.01%). Duo paternity (motherless) was the most common paternity test for both regular and court cases. We observed 367 paternity exclusions from 1135 cases, giving an overall paternity exclusion rate of 32.33%. Maternity had the lowest exclusion rate (8.33%), with criminal cases having the highest paternity (61.11%) and maternity (33.33%) exclusion rates. The number of mismatched STR loci ranged from 2–12 for duo cases and 4–18 for the trio cases. FGA, D2S1338, D18S51 and D2S441 were the most informative markers for exclusion. We detected 30 mutations out of 837 cases with an estimated paternal and maternal mutation rate of 0.0021 and 0.0011 respectively. Triallelic patterns were only observed at the TPOX locus with allele 10 and 11 being the extra alleles transmitted. Our report provides forensic parameters which can improve parentage and forensic analysis in Zimbabwe.

## Introduction

Short tandem repeat (STR) markers are a powerful tool in forensic genetic analysis, parentage testing, kinship analysis and population genetic studies. They exhibit high allelic variability due to high rate of germline mutations. STR markers are stably inherited from parents to children despite being highly variable making them effective for human identification. Twenty CODIS Core loci are currently recommended by the FBI for forensic DNA and human identification analysis and testing. These include CSF1PO, D3S1358, D5S818, D7S820, D8S1179, D13S317, D16S539, D18S51, D21S11, FGA, TH01, TPOX, vWA, D1S1656, D2S441, D2S1338, D10S1248, D12S391, D19S433 and D22S1045. These loci are commonly used because of their high heterozygosity, discriminatory power, clearly defined repetitive units and simple amplification and detection using commercial kits.

Testing for parentage can be differentiated into a variety of cases including the direct paternity and maternity test, as well as the indirect tests (kinship) such as grandparentage, siblingship and avuncular tests. These tests have been applied to solve peace of mind (regular), legal and human identification cases. The legal tests are conducted to meet the needs of justice, hence are conducted under strict conditions. Regular cases are conducted at the request of private individuals, but under less strict conditions compared to the legal cases. Paternity cases are the most conducted tests especially in private laboratories and requests for motherless paternity are often made. The testing in all these cases is based on genetic polymorphism, associated with differences between individuals. The informativeness of genetic markers is traditionally measured through finding inconsistences in parent–child Mendelian rules of transmission in randomly chosen individuals.

Parentage testing follows Mendelian inheritance law where the child receives one allele from each parent. There are however instances where spontaneous mutations can lead to mismatches, complicating maternity, or paternity cases^1^. Several mechanisms of STR mutation have been described and strand slippage have been identified as the main pattern of STR mutation^2^. Single step mutations are the most widely reported in routine parentage testing^3^ while multi-step mutations rarely occur^3,4^. These mutations complicate analysis of parentage cases as they affect the paternity or maternity index, and in such cases it becomes important to use mutation rates instead of the routine allele frequencies for calculation^5^. It is therefore important to account for the possibility of gene mutations when considering parentage exclusions. Parentage calculations can also be influence by several genotyping irregularities which include the null alleles and triallelic patterns.

Triallelic patterns are three peak profiles observed at a single locus and like mutations occur in rare cases. Two identical peaks are expected per locus with homozygotes presenting a single peak while heterozygotes present 2 peaks. Triallelic patterns present as either three imbalanced peaks (type 1) or three peaks of balanced peaks (type 2)^6^. The type 2 pattern can either be a 1:1:1 pattern or 2:1 pattern. Therefore, the investigation of tri-allelic patterns can help characterize the somatic and germline mutation of genetic makers and facilitate the statistical interpretation of STR loci during forensic DNA analysis. This paper provides an overview and analysis of parentage case types, mutation rates and triallelic patterns observed during routine parentage testing over a period of ten years. We determined the frequency of mutation rates and triallelic patterns in the Zimbabwean population. These have been shown to vary in populations affecting interpretation of results in parentage and forensic analysis. We also investigated the frequency of STR markers involved in the parentage exclusion. This assists in the choice of appropriate selection of STR typing kits.

## Results

### Parentage case types

We collected 1303 cases during a 10-year period (2013–2023). Cases included paternity, maternity, sibling, avuncular and grandparentage tests (Table 1). These were used to solve various cases including parentage disputes, criminal cases, disaster victim identification and inheritance disputes. Paternity was the most common type of tests (87.37%) followed by kinship (7.01%) and maternity (5.62%). Kinship included all tests where an alleged biological relative was tested to determine biological relationship in the absence of the alleged father. Motherless paternity (duo) was the most common paternity test, both for regular peace of mind and court ordered tests. Maternity tests were the least ordered test. It was however the most common performed tests for identification of disaster victims with 34 out of 73 cases (46.57%). Sibling test accounted for 54.94% indirect paternity tests followed by avuncular (37.36%) and grandparentage tests (7.69%).

**Table 1:** Parentage case types and exclusion rates (significant difference between peace of mind and court cases).

Type of case	Cases by genotyping kit (n)				Cases (n)	Exclusion rate (%)
	Identifiler	Identifiler + Verifiler	Panglobal	GlobalFiler		
Paternity						
Regular cases (duo)	130	44	12	497	683	31.43
Regular cases (trio)	209	17	–	74	300	28.42
Court cases (duo)	2	–	3	78	83	44.04
Court cases (trio)	7	4	–	26	37	37.83
Disaster victim identification	13	–	–	–	13	38.46
Criminal	2	–	–	16	18	61.11
Maternity						
Regular cases	4	–	4	19	27	3.70
Disaster Victim Identification	18	2	–	14	34	2.94
Criminal	2	–	–	10	12	33.33
Deceased or absent parent						
Sibling test	9	–	–	46	55	44.90
Grandparentage tests	–	–	–	7	7	42.85
Avuncular tests (aunt/uncle)	8	–	1	25	34	26.47
Total	404	67	20	812	1303	

Of the total tests conducted 59.75% of the disputed offspring were male while 40.25% were female.

### Parentage exclusion rates

Exclusion rates differed per parentage and case type. We observed 367 paternity exclusions from 1135 cases performed, giving an overall paternity exclusion rate of 32.33%. Paternity cases for resolving crime having the highest exclusion rate followed by court ordered duo cases (Table 1). Regular trio cases had the lowest paternity exclusion rate (28.42%). The exclusion rate for regular cases was lower compared to the court ordered tests for both duo and trio cases. This pattern was the same in the regular cases where lower exclusion rates were observed in trio cases compared to the duos. Maternity cases had the lowest exclusion rate for all case types (8.33%). The exclusion rate was however high for criminal cases where the exclusion rate was 33.33% (4 out of 12 cases). High exclusion rates were also observed for the indirect parentage tests with the sibling test having the highest exclusion rate (44.90%). The overall parentage exclusion rate was 31.16%. There were no inconclusive maternity or paternity results. We however observed 6 inconclusive results out of 91 indirect parentage tests.

**Figure 1:**
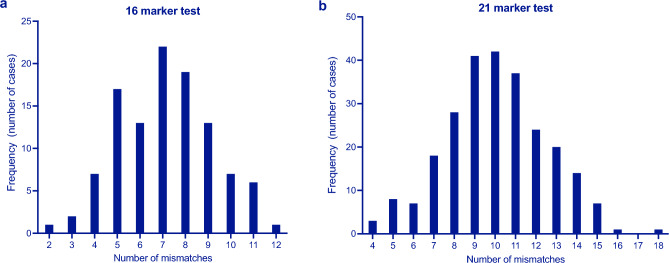
Number of mismatches observed in parentage cases where biological relationship was excluded. Cases were split into (**a**) 16 (n = 115) and (**b**) 21 marker tests (n = 251).

The number of mismatched STR loci for all cases ranged from 2–12 for duo cases and 4–18 for the trio cases. The analysis was only done for the direct parentage tests. The data was normally distributed as per Shapiro-Wilks test with α = 0.05 and p = 0.3265 and 0.0800 for the 16 and 21 marker test respectively. The mean of the distribution was 7 for the 16-marker test and 10.65 for the 21-marker test (Fig. 1). FGA and D2S1338 were the most frequent autosomal markers in parentage discrepancy (Table 2). FGA and D18S51 were the most frequent markers for duo cases while SE33 and D21S338 were most frequent for trio cases. D12S391 and D2S441 were the least informative markers in terms of exclusions in this population.

**Table 2:** Frequency of mismatches per each tested marker.

Marker	Duo (n = 262)		Trio (n = 102)		All cases (n = 364)	
	N	%	N	%	N	%
*D1S1656	110	52.38	25	58.14	135	43.27
D2S1338	175	66.79	69	67.65	244	67.03
*D2S441	66	31.43	18	41.86	84	26.92
D3S1358	93	35.50	61	59.80	154	42.31
D5S818	98	37.40	55	53.92	153	42.03
D7S820	108	41.22	60	58.82	168	46.15
D8S1179	107	40.84	52	50.98	159	43.68
*D10S1248	101	48.10	30	69.77	131	41.99
D12S391	91	34.73	9	8.82	100	27.47
D13S317	76	29.01	46	45.10	122	33.52
D16S539	105	40.08	57	55.88	162	44.51
D18S51	145	55.34	73	71.57	218	59.89
D19S433	144	54.96	63	61.76	207	56.87
D21S11	154	58.78	64	62.75	218	59.89
*D22S1045	100	47.62	24	55.81	124	39.74
CSF1PO	110	41.98	59	57.84	169	46.43
FGA	155	59.16	74	72.55	229	62.91
*SE33	143	68.10	35	34.31	178	57.05
TH01	80	30.53	38	37.25	118	32.42
TPOX	102	38.93	49	48.04	151	41.48
vWA	122	46.56	68	66.67	190	52.20

*Marker was not present in the 16 marker tests, therefore n = 210 for duos, 0 for trios and 312 for all cases.

### Triallelic patterns

We only observed triallelic patterns at the TPOX locus at a frequency of 0.0414. The three peaks encountered in all the cases were approximately of equal height confirming to the Clayton type 2 pattern^6^ except for one which was type 1 exhibiting all three uneven peaks. The majority of triallelic individuals had allele 10 (51 out of 54) except for 3 cases where the extra allele was 11. The observed triallelic genotypes are summarized in Table 3. The [8, 10, 11], [8, 9, 10] and [9, 10, 11] were the most frequent triallelic genotypes with frequencies of 0.1707, 0.1463 and 0.1707 respectively (Table 3). The rest of the genotypes were observed in relatively low frequencies. These were genotypes observed in all individuals with triallelic pattern regardless of whether they were related on not. A total of 60 unrelated individuals out of a total of 1089 were triallelic at the TPOX locus, translating to a frequency of 0.047. Of these 44 (73.33%) were type 2A while 16 (26.67%) were type 2B. The pattern was observed in 54 cases, where 52 were paternity, 1 maternity and 1 grandparentage case. Of the paternity cases 18 were exclusions and 34 inclusions. Both the maternity and grandparentage cases were inclusions. The inheritance pattern of the extra allele could not be conclusively determined since most of the cases were duo’s with either a missing mother or father. However, in 3 clear paternity inclusion cases, the fathers transmitted the extra allele to their daughters. Mothers on the other hand could transmit the extra allele to either their sons or daughters.

**Table 3:** Observed triallelic patterns at the TPOX locus.

Triallelic genotype	Number	Frequency
6, 6, 10	1	0.0122
6, 8, 10	5	0.0610
6, 8, 11	1	0.0122
6, 9, 10	1	0.0122
6, 10, 10	1	0.0122
6, 10, 11	5	0.0610
7, 8, 10	3	0.0366
7, 9, 10	1	0.0122
7, 10, 11	1	0.0122
8, 8, 10	7	0.0854
8, 9, 10	12	0.1463
8, 10, 10	3	0.0366
8, 10, 11	14	0.1707
8,10, 12	2	0.0244
8, 11, 11	1	0.0122
9, 9, 10	2	0.0244
9, 9, 11	1	0.0122
9, 10, 11	14	0.1707
9, 10, 12	1	0.0122
10, 10, 11	1	0.0122
10, 11, 11	5	0.0610

### Mutation rates

A total of 30 mutations were observed in the analyzed 837 cases (Table 4). Paternal mutations were more common as compared to the maternal mutations. Of the cases, 9 were mother–child pairs while 17 were paternal, where 11 were father-child duos and 6 mother-father-child trios. Mutations were observed in 12 of the 23 loci. Twenty-three of the cases (76.67%) were single step mutation events, while 2 step accounted for 6 cases (6.67%). The only 6 cases of the cases involved the loss of repeats while the remaining 24 were gain of repeats. Higher mutation rates were observed in loci with longer uninterrupted repeats for example FGA, D5S818 and SE33. The shorter ones D10S1248, CSFPO, D1S1656 and D2S244 had the lowest rates. The paternal mutation rate was 0.0021, while the maternal rates was 0.0011. The average mutation rate estimated across all loci was 0.0036. Mutations were not observed for Penta D, PentaE, TPOX, TH01, D16S539, D8S1179, D22S1045, D13S317, D7S820, D12S391 and D6S1043.

**Table 4:** Observed mutations for 21 STR loci in the Zimbabwean population (n = 837).

Locus	Paternal mutations			Maternal mutations			*Combined mutations	
	Allelic transfers	Mutations	Mutation rate	Allelic transfers	Mutations	Mutation rate	Mutations	Mutation rate
CSF1PO	770	0	0	770	1	0.0013	1	0.0013
D10S1248	527	0	0	527	0	0	1	0.0019
D19S433	770	2	0.0026	770	1	0.0013	3	0.0039
D1S1656	527	1	0.0019	527	0	0	1	0.0019
D21S11	770	1	0.0013	770	2	0.0026	3	0.0039
D2S1338	770	0	0	770	1	0.0013	2	0.0026
D2S441	527	0	0	527	1	0.0019	1	0.0019
D3S1358	770	2	0.0026	770	0	0	2	0.0026
D5S818	770	3	0.0039	770	0	0	4	0.0052
FGA	770	4	0.0052	770	2	0.0026	7	0.0091
SE33	479	3	0.0063	479	0	0	3	0.0063
VWA	770	1	0.0013	770	1	0.0013	2	0.0026
Overall	8220	17	0.0021	8220	9	0.0011	30	0.0036

*Includes 4 cases with mutations which could not be determined to be either maternal or paternal.

## Discussion

This is, to our knowledge, the first comprehensive report describing cases analyzed in a Zimbabwean paternity testing laboratory. We analyzed data from 1303 parentage cases in the over a 10-year period from 2013 to 2023. The cases were divided into paternity, maternity, grandparentage, sibling and avuncular tests. Paternity tests were the most popular test with the indirect tests being the least. The number of mismatched loci in excluded cases ranged from 2–18 depending on the testing kit with FGA and D2S1338 were the most frequent autosomal markers in parentage discrepancy.

Parentage testing plays an important role in determining biological relatedness, and the results have an impact in social, medical, judicial and immigration decisions. Paternity remains the most popular parentage tests and, in this study, it accounted for 87.02% of the requested tests. Most of the requested tests were for peace of mind (regular). The motherless paternity cases were the most frequently requested and conducted case despite its lower statistical weight as compared to the trio. Some of the reasons why it remains a popular test include affordability, privacy where the father does not want the mother to know of the tests and the fact that many of the cases were regular (non-legal) tests where the participation of the mother was optional. Some countries however discourage the motherless paternity tests to reduce the risk of false inclusions especially in cases where fewer STR markers are used, and the case background is not known^7–9^. As expected, maternity tests were not popular for solving parentage disputes. They were however the most requested tests for disaster victim identification as it is always assumed mother of a child is known especially where no criminal activity is suspected.

We observed an overall parentage exclusion rate of 31.16%. The paternity exclusion rate was 32.33 which is comparable to our previous findings^10^ but slightly higher than other reported rates^9,11–13^. This could be because exclusion rates were estimated from cases that arose from doubts regarding biological parenthood. Variations in exclusion rates are expected as they are influenced by factors such as sample size, the choice and number of STR markers used, societal factors and the population analyzed. There was a difference in the exclusion rate between the regular and court directed paternity cases (Table 1), with court cases having a higher exclusion rate. This could be explained by the fact that fathers who contest child support in courts are more confident of an exclusion result. Zimbabwean courts consider the welfare of the child first and is often in favor of granting child support. This therefore becomes an encouraging factor for paternity testing especially where the alleged father strongly believes the child is not theirs. The same is true for criminal cases where in many instances parentage testing was used as supporting evidence in kidnapping or late reported sexual assault cases. Exclusion rates were also high for indirect parentage tests where sibling tests had a rate of 44.90% while the rate for sibling tests was 42.85%. Most of the cases were used to settle inheritance disputes and involved children who were introduced to families after the death of one or both parents. This could be an indication of inheritance fraud or may be due to parenthood uncertainties especially in children born out of wedlock.

The usefulness of a genetic marker is measured by its power of exclusion, that is its ability to exclude the random man^14^. The average number of STR markers which determined the exclusion of paternity was 7 and 10.65 for the 16- and 21 marker tests respectively (Fig. 1). The most informative markers for exclusion across all cases were D21S338 (67.03%), FGA (62.91), SE33 (57.05%) and D18S51 (59.89%). In addition, D10S1248 was an important marker for exclusions in duo cases. This is consistent with reported power of exclusion in the Zimbabwean population where the same markers had the highest power of exclusion with D21S338, SE33, D10S1248, D1S1656 and Penta E having a power of exclusion of 0.7976, 0.8683, 0.7323, 0.7452 and 0.8064 respectively^15^. We could not determine the power of exclusion for Penta E in this study because of the small sample size. Of the total tests conducted 59.75% of the disputed offspring were male while 40.25% were female. This is comparable to our previous report^10^ and could further indicate stronger interest by families to claim or ascertain paternity of a male child^16^. Given that the male to female ratio at birth is 1.02 we therefore expect the number to be skewed towards more female children being tested. This ratio also declines with age, favoring the females, therefore there are more females in any group. Zimbabwe is a patrilineal society where descent is traced through the male line. Male children are therefore still considered superior to female children as they ensure the continuity of the family legacy and name^17^.

We only observed the triallelic genotype at the TPOX locus (Table 3). A threshold of 300 rfu was set to ensure the observations were not due to stochastic effects such as allele drop in, sister allele imbalance and elevated stutter. In addition, the data was generated from single source samples with adequate DNA concentrations to minimize the stochastic effects. Triallelic genotypes are rare but are expected to be encountered at all traditional regions used in forensic DNA analysis^6^. This has implications for paternity index calculations, with new methods being formulated according to the generation and genetic transmission of tri-allelic pattern^18^. All the triallelic genotypes observed in this study except for 1 were type 2 suggesting chromosomal duplication or aneuploidy where multiple alleles with peaks of equal height are produced^6^. Only one case had a type I pattern where all the three peaks were of unequal height. Type 1 mutation at the TPOX locus is rare^19^ and suggests somatic mutation at a heterozygous locus during development causing mosaicism, where some alleles have the mutant allele while others have the original allele^6^. The bias towards the type 2 allelic pattern was excepted and has been previously reported as biased toward type 2^20,21^. High frequency of triallelic patterns at the TPOX locus have also been previously reported^20^, with variation between populations and is highest among Africans^22^. Triallelic genotypes at other loci, for example TH01 and CSFPO have been reported but at very low frequencies^20,21^.

We observed an TPOX triallele frequency of 0.0414 which compares well with 0.024 in the South African population^23^. Frequencies ranging between 0.004 to 0.045 have been also reported in African populations^24^ while non-African regions have reported frequencies below 0.006^21,25,26^. The extra TPOX allele was allele 10 in most of the cases (51 out of 54) except for 3 cases where the extra allele was 11 (Table 3). The extra allele has been hypothesized as being a translocation of allele 10 onto chromosome X^23,25^. This agrees with our observations where fathers transmitted the extra allele to their daughters only. Mothers on the other hand could transmit the extra allele to either their sons or daughters. The presence of the allele however does not determine the alleles it is transmitted together with as seen from the variety of observed triallelic genotype combinations (Table 3). The high frequency of TPOX triallelic genotype was also expected as it has been previous reported to occur at high frequencies in African populations with allele 10 being transmitted as the extra allele^23,27^. The extra allele 11 is mainly found in Chinese and Korean populations^23^ and its presence in our populations could be explained by either population admixture or mutations.

Paternal mutations were more common when compared to maternal mutations and this is consistent with published literature. The paternal mutation rate was 0.0021, while the maternal rates was 0.0011. The average mutation rate estimated across all loci was 0.0036. Our mutation rates were higher compared to other populations^28–30^. Higher mutation rates were observed in the loci which are longer and have uninterrupted repeats for example FGA and SE33 (Table 4). This has been previously reported^31,32^. The mutation rates tended to differ between loci. This could possibly be due to replication slippages and stalling which tend to be more pronounced in longer repetitive sequences^33^. This however generates a high variety of polymorphisms resulting from gain or loss of alleles^34^. Mutations were not observed for Penta D, PentaE, TPOX, TH01, D16S539, D8S1179, D22S1045, D13S317, D7S820, D12S391 and D6S1043. This could be because of the small sample size as mutations rates for some STR loci have been reported to be low^5,28,32^. The mutation rates were corrected for the possibility of null alleles. We used a simple intuitive method where p was based on the count of null alleles inferred from parentage analysis where there was a mismatch between parent and child homozygous genotypes at the focal locus only. The frequency was then estimated as the number of inferred null heterozygotes divided by 2N assuming the null allele in a sample of N diploid individuals is rare. This was then used to correct the mutation frequencies.

## Conclusion

This study provides useful insights and data on parentage in Zimbabwe. Parentage testing is slowly gaining popularity in Zimbabwe. The number of tests carried our per year in the country is still very low compared to other countries. Some of the data and rates in this report are approximations and preliminary due to the small sample size. This is however the first comprehensive report on parentage testing data for Zimbabwe and provides useful information for further studies.

## Materials and methods

### Information dataset

The information dataset was obtained from cases that were performed over a 10-year period at the African Institute of Biomedical Science and Technology DNA testing center. All methods were performed in accordance with the relevant guidelines and regulations as set by the Health Professions Authority of Zimbabwe and the Medical Laboratory and Clinical Scientist Council of Zimbabwe (MLSCZ). Written consent to collect samples and conduct testing was obtained from the participants through an approved client identification form. The study experimental protocols were approved by the Research council of Zimbabwe (MRCZ/B/1323). The study was conducted following the ethical principles outlined in the Declaration of Helsinki. We created a database in excel where personal information of individuals involved in the paternity cases was not shared according to legal regulations (personal data protection, Zimbabwe). The laboratory conducts quality control proficiency annually organized by the English working group of the International Society for Forensic Genetics (ISFG).

### Genotyping

DNA from blood, buccal swabs or spotted FTA cards was extracted using either Prep-n-Go™ Buffer (Applied Biosystems by Life Technologies, UK) or standard extraction kits as per manufacturer’s instructions. PCR amplification was conducted using the following commercial HID kits as per manufacturer’s instructions: AmpFlSTR^®^ Identifiler^®^, GlobalFiler™ PCR Amplification Kit and VeriFiler™ Direct PCR Amplification Kit (Applied Biosystems by Life Technologies, UK) and SureID^®^ PanGlobal Human DNA Identification Kit (Health gene technologies). The kits were gradually introduced into the lab for paternity testing resolution. The amplified PCR product was separated by capillary electrophoresis on the 3500 Genetic analyzer and data collected using Data Collection v2 Software (Applied Biosystems). The Genemapper^®^ v 1.4 and corresponding allelic ladders were used for allele calling.

### Parentage analysis

We performed parentage analysis using The Mass Fatality Identification System (M-FISys) (GeneCodes, Michigan, USA). Paternity was called following published guidelines^35^. At least 15 loci were genotyped. Paternity was calculated at each STR locus as a likelihood ratio. This was generated by comparing the probability that the alleged father contributed the obligate allele with probability that the randomly chosen man contributed the allele. The combined paternity index (CPI) was calculated by multiplying the PI values at each locus. The probability of paternity (PP) was calculated using the formula PP = CPI/(CPI + 1). Cases showing 4 or more excluding loci and a CPI of less than 10,000 were excluded. PI computation in the presence of isolated mutations were used with a corresponding mutation rate (μ) and power of exclusion (PE) as recommended by the American Association of Blood Banks (AABB) (PI = μ/PE). The stepwise model was considered where more than two mutation steps occurred. Relatives such as aunt, uncle, grandparents, or siblings were used to determine biological kinship in cases where the alleged father was missing.

### Triallelic patterns

Data was collected from unrelated individuals with triallelic patterns in the tested locus. All samples containing a triallelic pattern were confirmed by re-extraction and amplification with a different STR typing kit. Data and physical counting were done in excel where number of cases and allelic combinations were recorded. The transmission of the extra allele was investigated from family cases with true biological relationship at loci that exhibited the triallelic pattern. The pattern type was allocated based on the observed intensities on the electropherogram, with triallelic variants categorized as described by Clayton and co-workers^6^. Peak intensities were used to identify the pattern type where alleles with 3 imbalanced peaks were identified as type 1 and those with equal intensity being identified as type 2.

### Mutation rates

Mutations were investigated in all parentage non excluded cases by investigating paternal and maternal allelic transmissions (meiosis). A total of 837 cases consisting of 532 father-child (paternity duos), 238 mother-father-child (paternity trios) and 67 mother–child (maternity duos) transfers were included in the analysis. Cases favoring a biological parent–child relationship (LR (write in full when using for first time) > 1000) were chosen. Mutations were considered where there was evidence of biological parent–child relationship, but one or two loci failed to match. Mutations were confirmed by typing with a different kit following recommendations and guidelines^36^. The number of allelic transmissions used to calculate the mutation rate were specified for each marker since we used different commercial kits for the genotyping. Biostatistical analysis was carried out in M-Fysis. Mutation rates at each STR locus were calculated using the relationship:

$$Locus\, Mutation\, rate= \frac{number\, of\, mutations\, detected\, at\, each\, locus }{total\, number\, of\, meiosis\, obeserved\, at\, the\, locus} \times 100$$

## Data Availability

The data that support the findings of this study are available from the African Institute of Biomedical Science and Technology (AiBST), but restrictions apply to the availability of this data, which was used under license for the current study, and so are not publicly available. Data is however available from the authors upon reasonable request and with permission of the African Institute of Biomedical Science and Technology (AiBST) and will be reviewed on a case-by-case basis.
